# Assessing the assessors: investigating the process of marking essays

**DOI:** 10.3389/froh.2024.1272692

**Published:** 2024-04-19

**Authors:** Adam Hasan, Bret Jones

**Affiliations:** ^1^ Centre for Dental Education, Faculty of Dentistry, Oral and Craniofacial Sciences, King’s College London, London, United Kingdom; ^2^ College of Engineering, Computer Science and Construction Management, California State University, Chico, CA, United States

**Keywords:** assessment, marking practice, criteria, grading, holistic marking

## Abstract

Pressure for accountability, transparency, and consistency of the assessment process is increasing. For assessing complex cognitive achievements, essays are probably the most familiar method, but essay scoring is notoriously unreliable. To address issues of assessment process, accountability, and consistency, this study explores essay marking practice amongst examiners in a UK dental school using a qualitative approach. Think aloud interviews were used to gain insight into how examiners make judgements whilst engaged in marking essays. The issues were multifactorial. These interviews revealed differing interpretations of assessment and corresponding individualised practices which contributed to skewing the outcome when essays were marked. Common to all examiners was the tendency to rank essays rather than adhere to criterion-referencing. Whether examiners mark holistically or analytically, essay marking guides presented a problem to inexperienced examiners, who needed more guidance and seemed reluctant to make definitive judgements. The marking and re-marking of scripts revealed that only 1 of the 9 examiners achieved the same grade category. All examiners awarded different scores corresponding to at least one grade difference; the magnitude of the difference was unrelated to experience examining. This study concludes that in order to improve assessment, there needs to be a shared understanding of standards and of how criteria are to be used for the benefit of staff and students.

## 1 Introduction

Growing pressures for accountability, transparency, and consistency from universities, government and from potentially litigious students are driving the need to account for validity and reliability in assessment (1). The increase in undergraduate fees further heightens the need for a robust assessment process (2). The quest for reliability can, however, skew assessment away from judgements of complex learning towards the assessment of simple and unambiguous achievements (3). Considerations of cost add to the skew towards assessment of what is easily measured and reliable, for example, the multiple-choice-question (MCQ) format, but which is a poor indicator of the candidate's higher order skills, professional-level judgement, and cognitive achievement (4).

Assessment has an effect on curriculum coverage. There is also a relationship between assessment and the way in which the subject is presented in teaching (3, 5). This in turn affects, through the tasks in which the students engage, what and how the students learn (5). In dental surgery, we would wish candidates to develop higher order skills, analytical skills, application of knowledge, investigation, reasoning and interpretation. Assessment should reflect this expectation. Essays require students to select and synthesise information, and to demonstrate their practice knowledge and understanding (4). In order to improve reliability in essay marking it is important to know how examiners deal with exam manuscripts. Whilst there may be an argument for removing essays because of the inter-examiner variation in grade awards, this would remove a valuable part of the assessment, as the ability to synthesise information, construct arguments and apply knowledge in depth cannot easily be achieved in other assessment formats (6).

In order to improve the assessment process, it is important to understand the problems with essay marking from the perspective of the examiners’ process and experience, so that appropriate strategies can be employed to enhance assessment quality and reliability. Ecclestone (7) reminds us that few in higher education are well-informed about the literature on assessment, and whilst assessors may be experts in their own subject, they are not experts in assessment. This lack of expertise in assessment results in underlying skewing dynamics in marking practice being unrecognised and uncorrected (8, 9). In fact, how assessors mark student work is not well known. Recent qualitative and quantitative research to explore marking practice continues to reveal the complexity of these little understood assessment practices (10–12).

Essays are usually assessed in one of two ways, either by mark-remark procedures with different markers scoring the same piece of work (inter-rater reliability) or by the same marker marking the same pieces of work on different occasions (intra-rater reliability). Markers are affected by characteristics of the students: presentation, clear handwriting, gender of candidate and marker (13). Clearly there are inter-rater differences in marking and in assessor understanding of the (and of their own) marking process, all of which can impact on assessment. Marker training and the provision of scoring rubrics can enhance reliability (14, 15). Inter-examiner moderation is also crucial when multiple examiners are involved, but that topic lies outside the scope of this study.

This paper argues that attention needs to be focused on inter-examiner agreement. Performance assessment is highly subjective, as it relies on professional judgement. However, if the assessors are trained, provided with scoring rubrics, and given exemplars of performance for each grade, then inter-examiner agreement can be high (16, 17). Increasing the number of tasks can increase score reliability and enhance generalisability; the underlying assumption is that these traits are stable over time. It is known that when examiners mark essays, scores can vary widely, however it is not known how examiners score essay papers in dentistry.

This study posits the following research questions:

How do examiners assign scores to candidate's essays?

1. How do examiners make judgements whilst marking essays?
2. How do examiners interpret the marking rubrics in assigning candidate scripts to particular grade categories when marking essays?

Issues impacting on assessment outcomes are considered in this section.

### 1.1 Context

The research setting is a dental school in the UK, with a clear emphasis on maintaining a cutting-edge research profile. The principal function is the provision of teaching and learning support for dental students, although the emphasis is clearly on research to maintain a cutting edge and thus an attractive image to prospective students. This has influenced staff to engage in research more readily and, unfortunately, to withdraw from teaching including the assessment process. The poor rating in assessment and feedback given by students in national surveys (18) supports the researcher's impression that assessment is not given the importance it deserves particularly in this era of accountability. The management structure is hierarchical with all staff taking part in formative and summative assessments. The staff involved in the examination process include both junior and senior members of staff from all subject disciplines.

There are over 80 students in Bachelor of Dental Surgery (BDS) year 4, and an examining team of 9. The examination process involves junior and senior members of staff from relevant subject disciplines. This study focused on assessors marking a task on the role of medical histories and radiological assessment in periodontal management. This assessment research is valuable to the dental school because it will (1) help to ensure candidate performance is appropriately rewarded (2) fill a void in the literature on essay marking in higher education in dentistry (3) enable summative assessment to be improved. As the final exams enable registration with the General Dental Council (GDC), the implications of this research could have a positive impact on the public.

## 2 Methodology

This study employs a mixed-methods approach: a quantitative approach for numerical data and score analysis and a qualitative approach to analyse the textual data and examiner process. This article concentrates on the qualitative aspects, using an interpretivist paradigm for analysis (19). An interpretivist paradigm assumes social reality is embedded in context thus enabling the exploration of how all nine examiners in this team use essay plans (rubrics) to assign scores to scripts (19).

**Figure 1:**
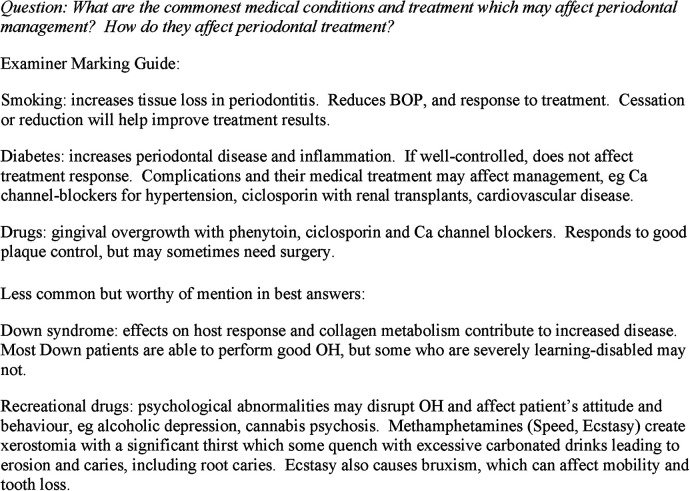
Assessment question and examiner marking guide.

For both research questions “think aloud interviews” (in which a participant verbalises his/her thought processes while working on a task) are used to investigate how examiners make judgements whilst marking essays (20). This technique helped to recruit all examiners in this team, and allowed them not to disrupt their routines or create additional burdens. This abrogates the need to engage in a post-marking, retrospective reflection/interpretation or to schedule more time-consuming semi-structured interviews in which the interviewees try to recall their experiences of marking in some detail. The assessment question and examiner marking guide is provided in Figure 1. The consistency in marking was also explored by asking examiners to re-mark anonymised essays they have previously marked. An interval of 9 weeks was selected so as not to interfere with academic duties and to maximise the period between the first marking and remarking before staff took leave. In order to compare how consistent the scores and grades were at the time of marking and again after an interval of 9 weeks, a paired t test was used. The answers will help to develop an assessment process that is more reliable and a closer reflection of candidate performance.

### 2.1 Recruitment

The researcher approached examiners individually and invited them to take part; in this way only the researcher knew who the participants were. In order to encourage participation of the entire team of 9 examiners who are academics with heavy teaching and clinical loads, it was crucial to employ methods that are time-efficient and easy to undertake; participants are therefore asked to record either in a written or oral form how they go about marking essays. This interview is conducted in the most convenient location for the participant, his/her office. The researcher does not use group interviews as group interview or focus groups are not designed to yield individual deep reflective data (21).

### 2.2 Ethical approval and issues in conducting this research

Informed consent was obtained from all participants. Participant identity is protected by anonymising the audio-tapes and coding any names in transcripts. The identity of students and staff is protected by using codes in the exam results. No year is indicated in the tabulated results, so that neither staff nor candidates from the cohort can be identified from tables of results. Ethical approval was obtained to carry out this study from King's College London (BDM/09/10-83) and from the Director of Education.

### 2.3 Analysis

The audiotapes were transcribed by a trained transcriber and verified by the researchers (Adam Hasan, Bret Jones) using an interpretativist approach (19). The researchers examined the transcripts and mapped understandings shared and not shared by respondent. The researchers coded the interview data, creating open codes as the transcripts are read (22). As codes were accumulated, the researchers re-coded to ensure there is consistency in coding transcripts, and then began to sort coding into themes (22, 23). Examiners reviewed their own transcribed comments and agreed their validity. No amendments were needed to the text. Nine Examiners were asked to re-mark essay scripts, so that we could compare consistency in assigning scores to previously marked scripts. These findings helped triangulate data, thereby gaining confidence in the interpretation of the responses.

## 3 Data presentation and discussion

The examining team consisted of 9 staff and all 9 members (E1–9) of the examining team consented to participate in this study.

### 3.1 Skewing of essay scores is multifactorial

Human resources, timing and use of the generic marking scheme (MS) all impact on assessment (24). Whilst most examiners use the essay marking guides when deciding scores, not all are using the generic MS which provides descriptors for each percentage band (Table 1). The descriptors are framed in comparative terms, and this may partly explain the tendency to mark within the range 30%–70% where the examiners can more readily interpret and apply better defined criteria (Table 1). However, as Price and Rust (26) have already found, having explicit criteria and grade descriptors does not improve the understanding of standards. This finding is also suggested by inconsistent essay marking in this study. The re-marking of scripts revealed that only 1 of the 9 examiners achieved the same grade category. All examiners awarded different scores, corresponding to at least one grade difference (6 of the 9 assessors), but sometimes revealing differences corresponding to 2 grade categories in 3 assessors (paired *t*-test, df = 8, *t* = 2.62, *p* < 0.05). The magnitude of the difference in scores was unrelated to experience examining (Table 2).

**Table 1:** Marking scheme grade descriptors (25).

Distinction 70+	*Thoughtful answer informed by wider reading showing clarity of thought and personal insight*	
	Understanding	thorough understanding demonstrated with an insightful and creative analysis
	Selection & coverage	comprehensive range of relevant evidence used, demonstrating independent study and extensive reading
	Structure	clear, fluent, integrated and focused
	Knowledge	Excellent level of knowledge, no inaccuracies
	General	90 + creative and sophisticated
		80 + striking insight demonstrated
		70 + excellent in all areas, displaying originality
Merit 60–69	*Good understanding of basic principles & relevant evidence, with a coherent & logical argument showing analytical ability*	
	Understanding	good understanding of all key issues and wider implications with a convincing analysis
	Selection & coverage	breadth in examples and evidence used without any major omissions, evidence of extended reading
	Structure	coherent and logical
	Knowledge	Good, above average level of knowledge, minor inaccuracies only
	General	excellent in some areas or of high quality in all
Pass 50–59	*Sound understanding demonstrated*	
	Understanding	sound understanding of basic principles and main issues with some evidence of analysis or synthesis
	Selection & coverage	appropriate material but little evidence of extended reading, possibly some minor omissions
	Structure	clearly presented but little development of answer
	Knowledge	Average, acceptable level of knowledge, minor inaccuracies, no serious errors of fact
	General	lower quality answer in at least one area
Borderline Fail 46–49	*Basic understanding of the main issues demonstrated, too little information (NB. Could be compensated by other questions)*	
	Understanding	general knowledge demonstrated but analysis limited in depth and breadth. Safe but lacks some demonstrated knowledge.
	Selection & coverage	skeletal coverage of basic material, some omissions but not to detriment of a patient
	Structure	inadequately presented
	Knowledge	Inadequate level of knowledge shown. Significant inaccuracies, but none to detriment of patient care
	General	superficial and a low quality answer
Fail 0–45	*Unsystematic, incomplete and/or inaccurate*	
	Understanding	key issues not identified, poor analysis or none. Not safe to proceed with this level of understanding
	Selection & coverage	some inaccuracies or omissions, excessive inappropriate material
	Structure	argument sketchy, loose ends, disorganised
	Knowledge	Inadequate level of knowledge, inaccuracies shown that may be to detriment of patient care. Not safe to proceed.
	General	36–45 some knowledge but poorly presented. Not shown to be safe.
		26–35 answered only in part and flawed. Not shown to be safe.
		16–25 deeply flawed or unacceptably brief. Unsafe.
		1–15 irrelevant or unintelligible. Grossly unsafe. 0—totally inadequate or no attempt to answer question

**Table 2:** Summary of examiners and length of commentary (25).

EXAMINER	E1	E2	E3	E4	E5	E6	E7	E8	E9
Training in examining	No	No	No	No	No	No	No	Yes	No
Post-graduate Qualification in Education	Yes	Yes	Yes	No	No	No	No	No	No
Experience in examining (years)	10	3	5	1	9	30	1	1	34
Length of commentary	715	3,486	954	739	1,517	757	1,334	1,099	511

In terms of human resources, increasing numbers of examiners are needed to cope with larger cohorts of candidates, which deepens concerns for validity and reliability as examiners may inadvertently employ multiple sets of criteria (26). It is now acknowledged that local communities are less able to establish standards unless both explicit as well as tacit knowledge about the standards are transferred (8). Despite this, the Quality Assurance Agency, is the independent expert quality body for higher education across the UK (27), has attempted to set explicit standards, failing to recognise the importance of tacit knowledge in assessment. Without assessment standards and a shared understanding of criteria, consistency is less likely. This is magnified when there are multiple markers (8, 28).

Timing is linked to both MS and human resources issues, in that the skewing problem is not solved simply by minimising the number of examiner pairs in an attempt to avoid the problem of multiple sets of criteria. The potential for skewing still exists as a result of examiner exhaustion in large-scale marking, as does the potential for examiners simply to mark essays within the limited time available with little regard for the quality of assessment (8, 24). The researchers agree with Knight (21) who finds when there are increased pressures due to increased workloads and reducing resources; the impact can only be negative on assessment. One examiner voiced this pressure.

E3 There's no time to reread all, just do the first lot and then when I’m in the zone as it were, I crack on using then time I have, since we have such a tight turnaround time for these, I’d be thinking of how much time I have, then make it fit the number of essays I have to mark. It's not ideal but that's all I can do.

### 3.2 Positivist background and discomfort with subjectivity in marking

In most of the commentaries there is evidence, either explicit or implicit, of differing philosophical perspectives, with different examiners seeking different levels of “truth” and “correctness”. For some, the text can only have one meaning. E6, for example, was very clear about what was right and wrong within the script s/he marked.

E6 Some important points, DPT, not differentiating, important points. That's wrong-2 views at right angles….change the views for furcations.

E6 is a well-established researcher and clinician, and clearly has a positivist epistemological viewpoint where s/he judges candidate responses dichotomously as right or wrong, with no degrees of correctness. Other examiners also reveal positivistic perspectives, mentioning “bias” and the importance of avoiding bias. The strong clinical and scientific background of the examiners may contribute to a feeling of discomfort in the perceived subjectivity of some aspects of marking. However, for those with post-graduate qualifications in education, the distinction between correct and incorrect is less rigidly defined. This more nuanced marker perspective is reflected in the relatively vague judgement language they employ, as if they are trying to determine the candidate perspective. They are, in a way, engaging in a dialogue with the author, a dynamic also found in Crisp (29). This suggests that those examiners with post-graduate educational training may be able to mitigate the inappropriate application of positivist perspectives to complex and nuanced assessment tasks, such as essays. What is clear is that these differing philosophical perspectives lead to different assessment outcomes. The implication is that examiners need to be aware of and clear about their own implicit philosophical perspectives, conditioned by their background and training, as they approach their understanding of shared criteria.

### 3.3 Marking criteria are used to rank candidates thwarting criterion-referencing

Common to all examiners in this study, irrespective of the marking strategy used, is the comparison and ranking of one candidate's script in relation to another.

E6 This does not have the structure of the first one. It's a pass, 1–2 marks below the first one, 56–57.

Norm-referencing is grading in relation to other candidates and is perhaps inadvertently promoted by the generic criteria in the MS which are couched in relative rather than absolute terms. Norm-referencing occurs because in practice it is easier to rank than to measure against criteria or an absolute standard (8). However, it is inappropriate, as there will always be some candidates who lie within the top 4% but none may have reached the level of performance/achievement that is compatible with safe clinical practice, consequently standards may vary widely over time. Criterion-referencing is preferred because grading using criteria reflects individual achievement rather than the achievement in relation to other students in the cohort. This is particularly important when the safety of the public has to be protected.

All examiners are clearly ranking the scripts through comparison rather than criteria. This further supports the notion that the 0–100 scale does not work well with these examiners. E1 believes that marking cannot be accomplished with the precision implied by the 0–100 scale, citing the difficulty in distinguishing a score of 51% from 52%.

E1 I aim to give a range representing where I think the paper sits in the marking range and a specific mark, the range indicates how far I am prepared to move if the co-examiner has a different mark. I don’t really believe I can give a specific mark, and be able to distinguish between 51% and 52%, this seems quite ridiculous to me.

It seems that ranking the scripts is easier to do than applying criteria (8, 29). According to Lumley (30), if essay marking guides do not answer the examiner's queries about script marking, the markers then attempt to reconcile their impression of the script with the rating guidance. It is a finding that matches this study. Examiners resort to using the generic MS as well as essay marking guides or model answers, but do not always find the answers they seek. The essay marking guide differs from a comprehensive “model answer”, presenting a problem to the inexperienced examiners who are in need of more guidance in how to use criteria and make judgements.

E7 Looking at the model answer it doesn’t really give a lot of flexibility in delineating between the passes, goods and excellent. You’ve got extra additional marks for the cone beam CT and that's as much that would take somebody into a better category, but there isn’t a lot of guidance on there on what a pass would be, or what a good pass would be or…

There appears to be different conceptions of what criteria-based marking/grading means; all examiners in this study are applying their own individualised interpretations of assessment when marking. Agreeing with Price (8) and Sadler (31), we found examiners within the same team had different theoretical interpretations translating to related but different practices (8, 31). Unless the assessment values and local practice are shared amongst staff, there is little chance of understanding what is meant by criteria and how these are to be applied (8).

The use of criteria in essay marking enables assessment of an absolute, rather than a relative, standard to be determined; this helps improve reliability and helps to reassure the public that candidates have reached the required standard (32). Criterion-referenced assessment is supposed to be a low-inference procedure (13) because of the careful specification of the domain or construct and thorough sampling of it. The specification is necessarily detailed; however, if too narrowly defined the assessment criteria lead to fragmentation of the task and a proliferation of discrete assessment tasks (3).

It would seem that knowing more about assessment and holding post-graduate qualifications in education, as do E1–E3, is not enough to ensure consistency in marking practice. Only two, E1 and E2, explicitly refer to criteria in their interviews. E2 highlights the importance of clarity of marking criteria and how lack of it impacts on consistency.

criteria for marking because it should be criterion referenced when different markers… you don’t have clear cut criteria it's difficult to get consistency between markers. What I find important with essays is that you should have clear cut with essays as in they are extremely time-consuming and if you’re marking essay papers. I do find that there are some problems.

Designing criterion-referenced assessment is difficult, particularly in advanced levels, involving complex subject areas, because “as the requirements become more abstract and demanding so the task of defining the performance becomes more complex and unreliable” (3). Problems can arise if question items do not reflect the intended constructs (under-represented constructs). It will then tend to unidimensionality and measurement of a single underlying attribute. In-so-doing, the test measures part of the construct, rather than its multi-dimensionality. The resulting scores cannot then be broadly interpreted (13, 33). In other words, incomplete assessment of the construct compromises validity and risks failing candidates unnecessarily. The tacit knowledge characterising “expert” performance complicates determination of valid constructs.

It is interesting to note that none of examiners refers to assessment objectives at all. There is no shared understanding amongst this team of examiners, but discernible confusion about how criteria should be used to determine standards. For many of the examiners in this study, there is implicit assessment of criteria, in order to determine the standard; however there is a lack of clarity between what is meant by criteria and standards, a finding that echoes Sadler (31). For some inexperienced examiners, determination of the higher passing levels is achieved by rigidly adhering to the essay marking guide; however, their consistency in determining this standard seems compromised by not knowing how to assess the additional contextualisation and quality of responses provided by better candidates. The net effect is reliance on other criteria which are not in the essay marking guide or marking scheme:

E7 It's still ok but still they haven’t brought a lot in CBCT aspect, but it's a fairly ok answer. It's got most of the information. Doesn’t really have a beginning and an end it's just somebody who has thrown all the information on the paper which is easy to pick up but it's very different from that first one. And it's got the same, less than the last one. I’d say it's a very safe pass, 58–59.

The only criterion directly relevant to the notion of standards in clinical practice is safety, as mentioned by all examiners, and is used to distinguish pass/fail. However, this criterion alone is unlikely to be sufficient to compensate for the diverse range of examiner marking practices but rather adds to the numerical skewing described earlier.

### 3.4 Holistic or analytic approach to essay marking?

The holistic scoring method is based on the theory that a whole piece of writing is greater than the sum of its parts (6). In this practice, essays are read for the total impression they create, rather than for individual aspects. The assessor is not overly concerned with any one aspect of writing, but looks at the response as a whole. The markers in this study could be categorised crudely as one of two types:

Analytic-like; or

Holistic-like

There are 3 examiners, E2, E3 and E8, who appear to be marking using an analytic framework, whereas the others are marking using a general impression system or holistic scoring system. E2's analytic approach to marking is captured in this excerpt:

E2 “…so that I could have a checklist to see how many they had covered, and if they had were giving me accurate responses and they were current and up-to-date.”

E2 and E3 are more systematic in their approach creating notes or a mental checklist as they assess scripts. Amongst those who have a “checklist” approach most appear to look for the content and level of understanding reflected in the candidate response.

E2…has looked at the use of anaesthetics treatment of the patient sitting upright and also has discussed that the patient would bring their inhaler to the appointment and that would be close by, and avoid the use of rubber dam to avoid further obstruction of the airway so she has covered some of the main points. I would have liked may be a little more description about asthma to begin with. I do like if they define what it is in a little bit more detail just to show that they understand.

E8 places a greater emphasis on identifying a relevant word. Although s/he uses checklists, E8 does not determine the word's presence, context and the level of understanding in a “tick-box” strategy for marking.

E8…they’ve mentioned the possibility of asthma and inverted commas decreased lung function. They actually haven’t said that asthmatics should have their inhaler so that should be something that should be mentioned it's not.

There are 5 examiners who are looking for content and evidence of understanding without necessarily being concerned if some elements are missing as long as the scripts reveal higher order skills, whilst the others focus on content, not understanding. Although most examiners summarise the strengths and weaknesses before coming to the final mark, it is not clear how the final mark is determined. For some inexperienced examiners, determination of the higher passing levels is achieved by rigidly adhering to the essay marking guide; however, their consistency in determining this standard seems compromised by not knowing how to assess the additional contextualisation and quality of responses provided by better candidates. These examiners prefer bullet points, where there is less burden, as the volume of text is simpler and limited. When such conflicts arise, the examiners tend to rely on criteria which are not in the essay marking guide or MS, such as sequence of responses and development of argument in the essay.

E7 It's still ok but still they haven’t brought a lot in CBCT aspect, but it's a fairly ok answer. It's got most of the information. Doesn’t really have a beginning and an end it's just somebody who has thrown all the information on the paper ….

E7's and E4's discomfort in making judgements is evident in their commentaries as well as in their reluctance to make firm decisions and to compensate for deficiencies in the essay marking guide. The need for professionalism in assessment has recently been noted (11). The lack of clarity on how to proceed and deal with these issues impact on the final mark, and eventually leads to different ways of coping with the percentage scale and essay marking guide that form a poor fit.

E7…it's very different from that first one. And it's got the same, less than the last one. I’d say it's a very safe pass, 58–59.

It is interesting to note that, E2, an examiner with a post-graduate qualification in education shows a clear understanding of what norm-referencing is, acknowledges that it is inappropriate in criterion-referenced marking, nevertheless E2 employs norm-referencing by ranking candidate's essays. Clearly, the criteria are themselves not enough for E2 who also needs to know how to apply the criteria to establish the standard. Some examiners solve this difficulty by counting the number of correct responses in relation to the essay marking guide before deciding on the grade and mark, rather than considering relevance and importance to the essay.

The more experienced examiners or those with qualifications in education determine the grade first then assign a score, whereas the inexperienced examiners are attempting to assign a score then discover the difficulties of applying a 0–100 range, a point also noted in the literature (10). Although most examiners consider both strengths and weaknesses of a script, there seems to be a focus on omissions when determining the score. Although comparisons are clearly used to rank candidate scripts, they may be advantageous in reducing the possibility of marking harshly (34). There is, in addition, the “halo” effect where if good scripts are encountered after a series of weak scripts, they are given higher marks (35). The sequence of scripts, particularly if they represent extremes, affects examiner marking of subsequent scripts with examiners more likely to link future judgements to initial judgements (36). The differing approaches to handling and applying criteria coupled with an analytic or holistic strategy can only increase the possibility of divergent score assignment thereby adding to the skewing effect. Recently some researchers have proposed using marking parties as a means of reducing the tedium and inconsistency in marking (12).

### 3.5 Conclusions and recommendations

The purpose of this project is to improve assessment practice in this school, thereby helping to ensure that standards are maintained and that the public is protected. Grade profiles are more useful to learners than a single score, providing them with important clues as to how they can improve their performance. Analytic marking using model answers is prescriptive but facilitates feedback to candidates. However, the time constraints and the reduction of resources are likely to direct essay assessment to a holistic marking strategy even though this is less helpful to candidates. The greater flexibility inherent in holistic marking is more appropriate in higher education and this marking strategy need not be limiting if the marking employs carefully constructed grade descriptors.

Standards and criteria need to be shared with staff in order to facilitate their internalisation by staff for assessment, and by students for improved future achievement. The assessment paperwork can be modified to complement the assessment process, so that there is correspondence, rather than incongruence, between criteria of the generic MS and the essay marking guides. This can be achieved by linking the generic criteria, and by linking specific criteria to the mark sheets so that the examiners can clearly see how the criteria and standard descriptors are linked. This in itself may be insufficient, and adequate time needs to be invested into assessment to develop and disseminate shared understandings of standards and practice so that the assessment process for a given cohort is valid and reliable. This point, particularly, is important for both inexperienced and experienced examiners as this study shows that experienced examiners can themselves skew results with their individualised sets of criteria.

Essays are creative pieces of work. No essay plan or model answer can anticipate the range of candidate responses; often the responses will not fit neatly into the categories created by the examiner. This problem can to some extent be alleviated by using exemplars to illustrate the standards in combination with the establishment of a consensual standard in academic communities. Without discussion about standards and exemplars to help in score assignment, the examiners simply resort to ranking, as they do in this study, giving secondary consideration to the percentage scale. The consequence is a narrower range of marks not employing the full scale.

Whilst this study has elucidated problematic issues in assigning scores, it has also uncovered areas which deserve further attention, including how examiners respond to essays emotionally and to essays of differing lengths; psychological aspects of judgements in essay marking; how positivistic or other philosophical stances influence marking; exploration of how other assessment scales can improve reliability in essays marking. In order to address the pressure on educators, examiners, and institutions, further study of these issues will raise the level of accountability, transparency, and consistency of the assessment process and help to provide assurance of reliable assessments. There is a need to study these issues further as well as how assessment process validity can be enhanced by helping assessors develop a shared understanding using a holistic approach with grade descriptors and exemplars.

## Data Availability

The raw data supporting the conclusions of this article will be made available by the authors, without undue reservation.
